# Supplementary material for: Ubiquitin ligase RNF20 coordinates sequential adipose thermogenesis with brown and beige fat-specific substrates
Source: Nat Commun. 2024 Jan 31;15:940. doi: 10.1038/s41467-024-45270-7 (PMC10831072; doi:10.1038/s41467-024-45270-7)
Supplement: Supplementary file 1 — Supplementary Information [file 41467_2024_45270_MOESM1_ESM.pdf]

# Supplementary Fig. 1 | RNF20 Deficiency Does Not Significantly Affect Brown Adipocyte Differentiation.

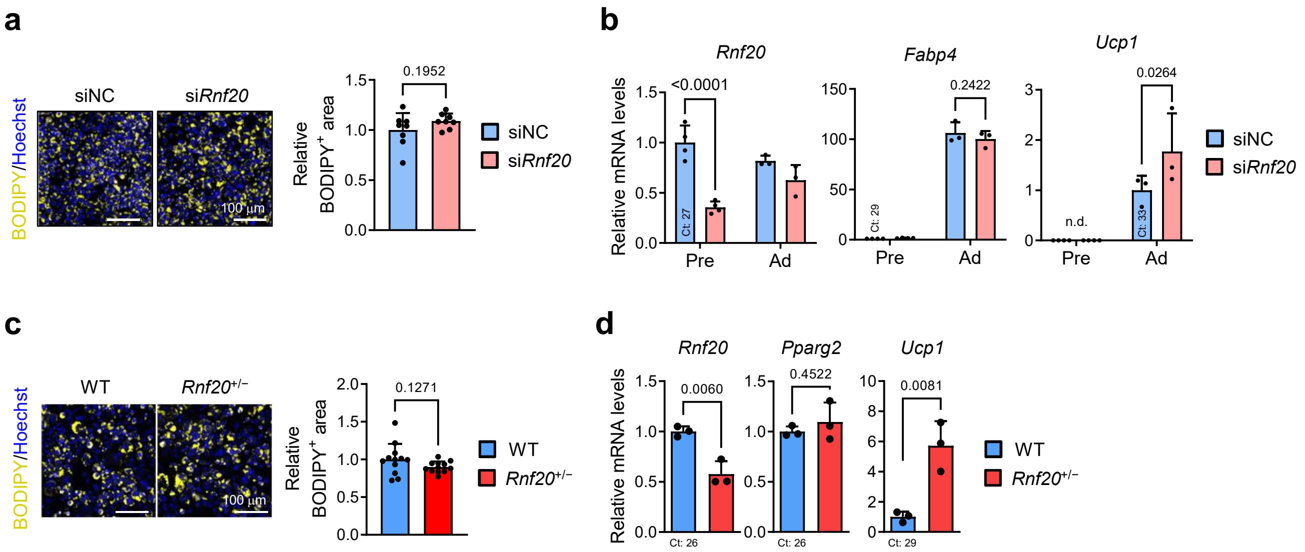


**Supplementary Fig. 1 | RNF20 Deficiency Does Not Significantly Affect Brown Adipocyte Differentiation. Related to Fig. 1. a**, (Left) Representative image of BODIPY lipid staining of differentiated brown adipocytes, BACs, transfected with siRNA against negative control (siNC) or siRNA against *Rnf20* (si*Rnf20*) before adipogenic induction. Scale bar: 100 µm. (Right) Quantitative analysis of BODIPY area per an image. n.d. not detected. **B**, qRT-PCR analysis of differentiated BACs transfected with siNC or si*Rnf20* before adipogenic induction. $n = 3$. Scale bar: 100 µm. **c**, (Left) Representative image of BODIPY lipid staining of differentiated SVF-derived adipocytes of BAT from WT and $Rnf20^{+/-}$ mice. SVFs were differentiated into brown adipocytes with differentiation cocktail. Scale bar: 100 µm. (Right) Quantitative analysis of BODIPY area per image. **d**, qRT-PCR analysis of differentiated SVF-derived brown adipocytes of BAT from WT and $Rnf20^{+/-}$ mice. All data are representative results from two independent experiments. $n = 3$. $n$ indicates biological replicates. Source data are provided as a Source Data file. Data are represented as mean ± S.D. Significance was determined using unpaired Student t-test (**a, b, d**) and two-way ANOVA (**b**).

# Supplementary Fig. 2 | RNF20 Is Downregulated in Thermogenic Brown Adipocytes.

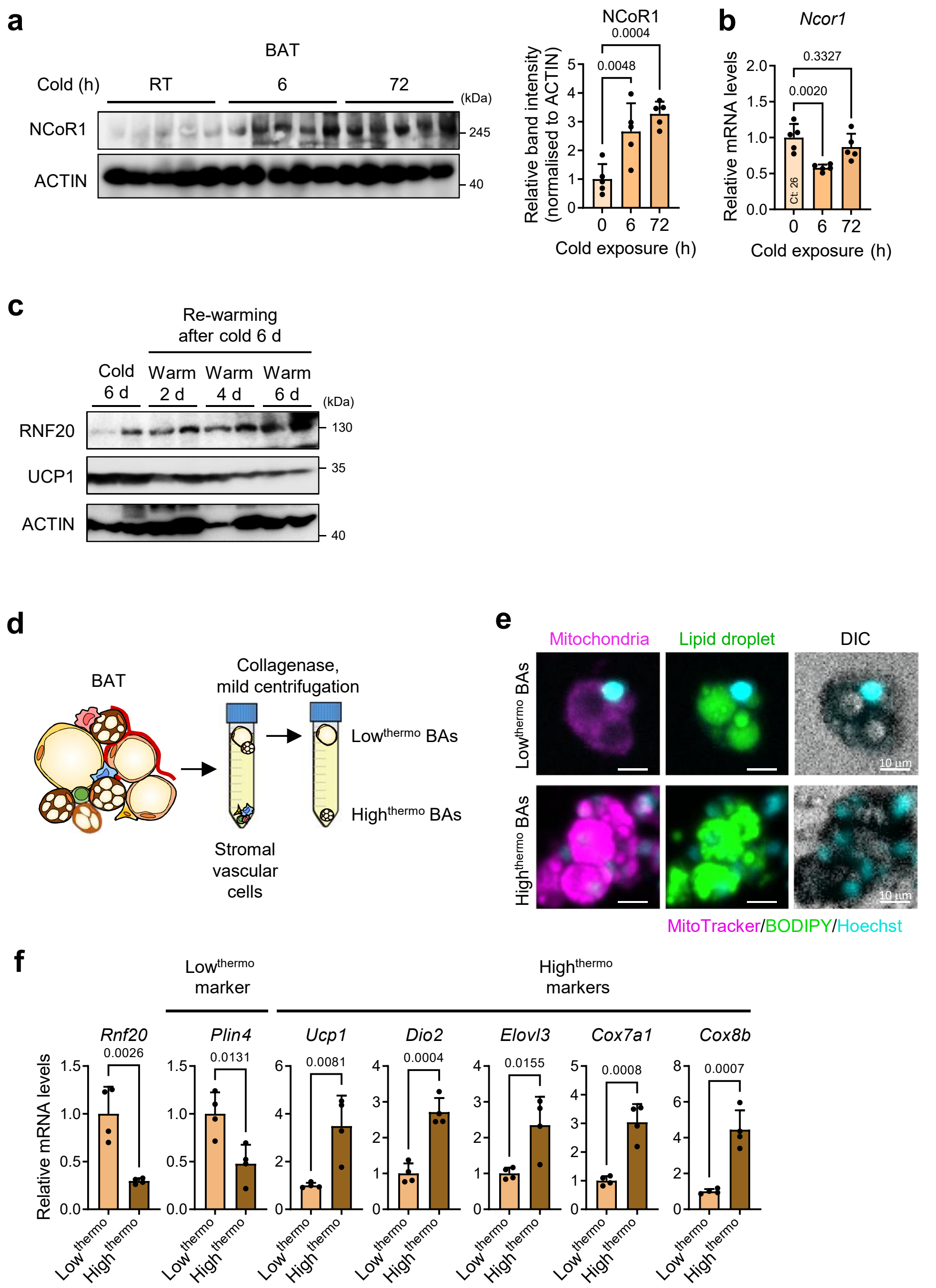


**Supplementary Fig. 2 | RNF20 Is Downregulated in Thermogenic Brown Adipocytes. Related to Fig. 1. a, b**, Protein (**a**) and mRNA (**b**) levels of NCoR1 in BAT of mice housed at room temperature (RT) and then exposed to cold (6°C) for 6 h and 3 d. **c**, Protein levels of RNF20 and UCP1 in BAT of mice housed at thermoneutral condition (TN, 30°C) and then exposed to cold (6°C) for 6 d. Then, mice were re-exposed to TN for 2, 4, and 6 d. **d**, Experimental scheme for sorting low and high thermogenic brown adipocytes-enriched fraction ($Low^{thermo}$ and $High^{thermo}$ BAs, respectively) from brown adipose tissue (BAT). **e**, Representative images $Low^{thermo}$ and $High^{thermo}$ BAs. Scale bar: 10 μm. **f**, qRT-PCR analysis of $Low^{thermo}$ and $High^{thermo}$ BAs. $n$ = 4. Source data are provided as a Source Data file. $n$ indicates biological replicates. Ct indicates critical value of qRT-PCR analysis. Data are represented as mean ± S.D. Significance was determined using one-way ANOVA (**a, b**) and unpaired Student t-test (**f**).

# Supplementary Fig. 3 | *Rnf20*$^{+/-}$ Mice Exhibit Enhanced Thermogenesis upon Cold Stimuli.

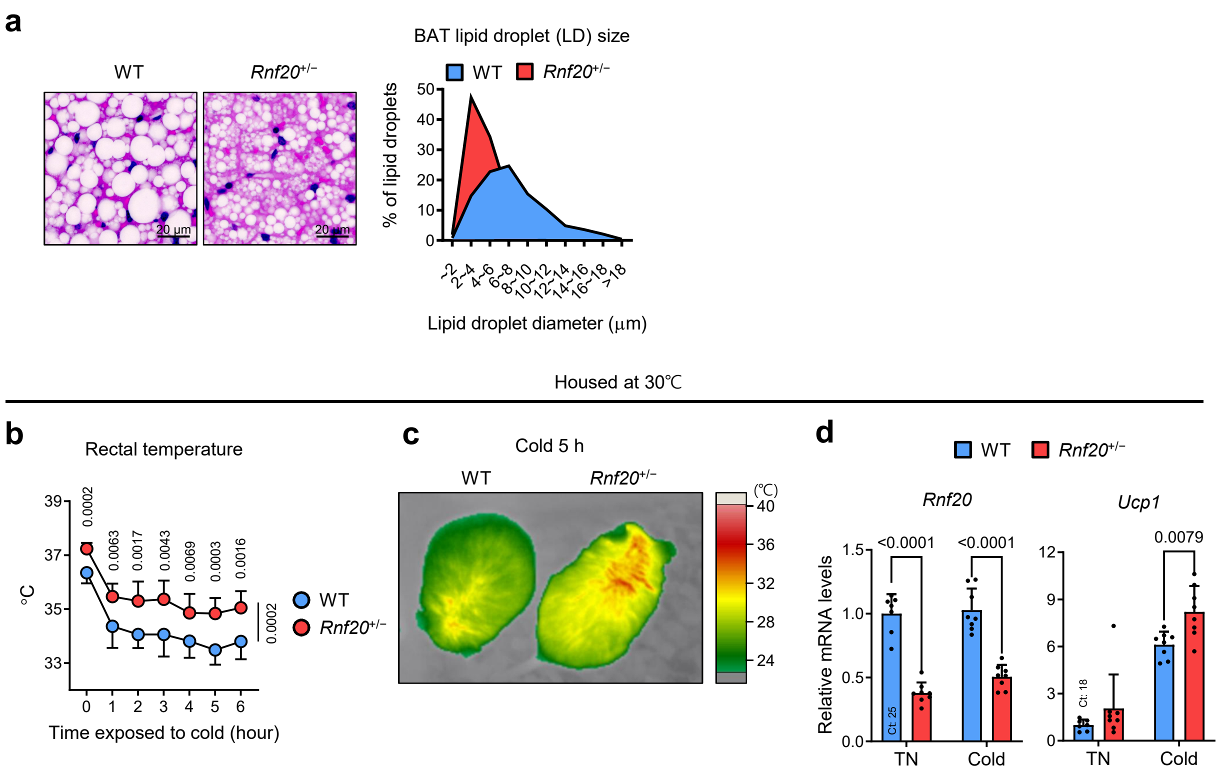


**Supplementary Fig. 3 | *Rnf20*$^{+/-}$ Mice Exhibit Enhanced Thermogenesis upon Cold Stimuli. Related to Fig. 1. a**, (left) Representative H&E staining of BAT of WT and *Rnf20*$^{+/-}$ mice housed at RT. Scale bar: 20 µm. (right) Distribution of lipid droplet (LD) sizes of BAT of WT and *Rnf20*$^{+/-}$ mice. Sizes of 500–600 LDs were measured in each group. **b**, Rectal temperature of WT and *Rnf20*$^{+/-}$ mice housed at TN (30°C) and then exposed to cold (6°C) . $n$ = 8. **c**, Representative infrared images of WT and *Rnf20*$^{+/-}$ mice housed at TN and then exposed to cold for 6 h. **d**, qRT-PCR analysis of BAT of WT and *Rnf20*$^{+/-}$ mice housed at TN and then exposed to cold for 6 h. $n$ = 7–8. $n$ indicates biological replicates. Source data are provided as a Source Data file. Data are represented as mean ± S.D. Significance was determined using repeated measures ANOVA (**b**) and two-way ANOVA (**d**).

# Supplementary Fig. 4 | Metabolic Phenotypes of *In Vivo* Overexpression or Knockdown of RNF20 in BAT.

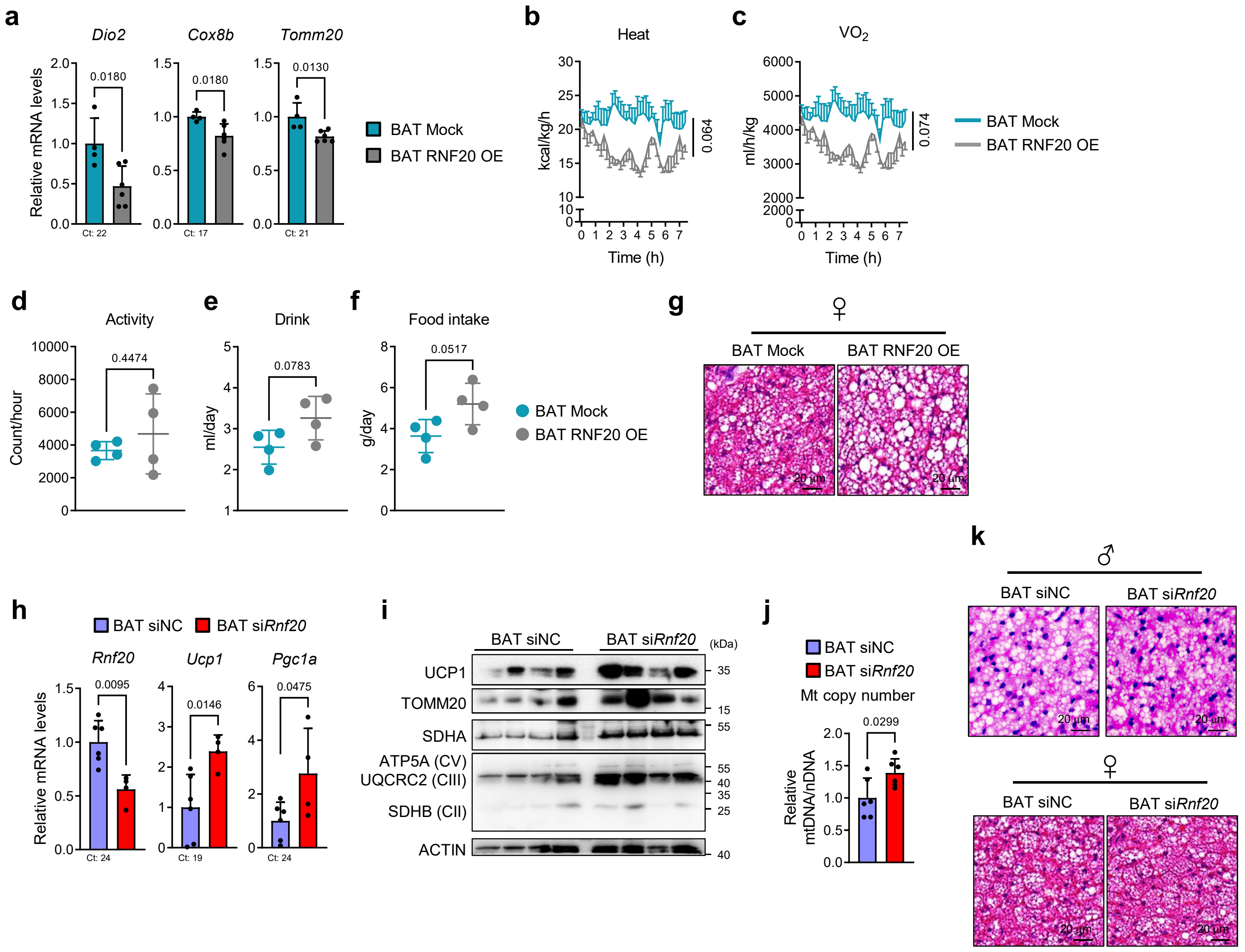


**Supplementary Fig. 4 | Metabolic Phenotypes of *In Vivo* Overexpression or Knockdown of RNF20 in BAT. Related to Fig. 2. a**, qRT-PCR of BAT from Mock and RNF20 OE mice. $n$ = 4–6. **b, c**, Wholebody heat generation and oxygen consumption of BAT mock and BAT RNF20 OE mice. $n$ = 4. **d–f,** Physical activity, drink, and food intake analysis of BAT mock and BAT RNF20 OE mice assessed by indirect calorimetry $n$ = 4. **g**, Representative H&E images of BAT from BAT mock and BAT RNF20 OE female mice. **h**, qRT-PCR of BAT from siNC and si*Rnf20* mice. $n$ = 4–6. **i, j** Western blotting analysis and mtDNA content of BAT from siNC or si*Rnf20* mice. $n$ = 6. **k**, Representative H&E images of BAT from siNC or si*Rnf20* male and female mice. Source data are provided as a Source Data file. $n$ indicates biological replicates. Ct indicates critical value of qRT-PCR analysis. Data are represented as mean ± S.D. Significance was determined using unpaired Student t-test (**a**, **d–f, h**, **j**) and repeated measures ANOVA (**b, c**).

# Supplementary Fig. 5. RNF20 Defects Drive Gene Expression Profile to Suppress ‘Whitish’ Features.

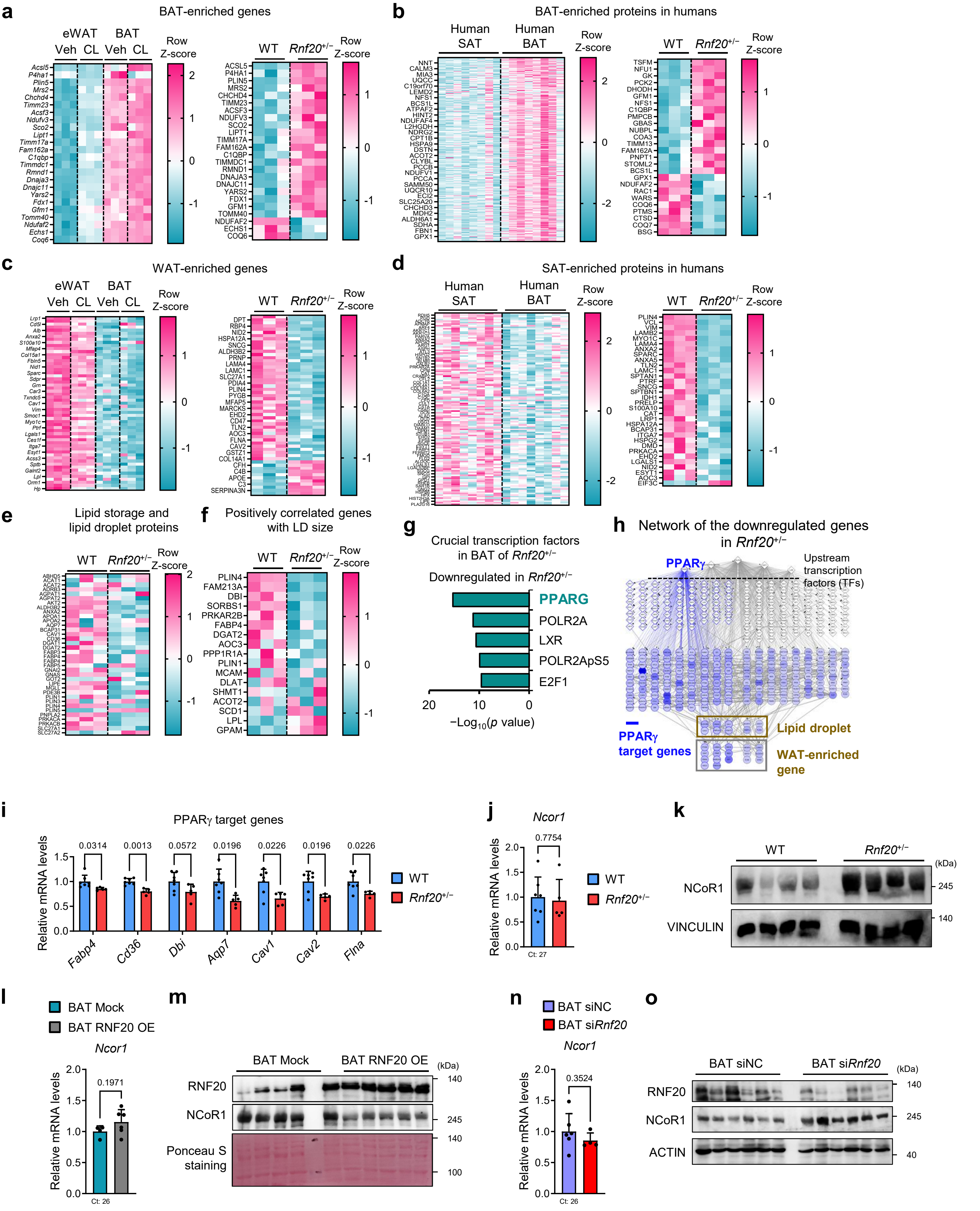

# Supplementary Fig. 5. RNF20 Defects Drive Gene Expression Profile to Suppress ‘Whitish’ Features.

**Supplementary Fig. 5 | RNF20 Defects Drive Gene Expression Profile to Suppress ‘Whitish’ Features. Related to Fig. 3. a,** (Left) Meta-analysis of epididymal WAT (eWAT) and BAT transcriptome data (Hepler et al., GSE98132). Heatmap showing relative expression levels of BAT-enriched genes. (Right) Heatmap showing relative protein levels of BAT-enriched genes in the proteome of WT and *Rnf20*$^{+/-}$ mice. Veh: vehicle treatment; CL: CL316,243 treatment for 3 d. **b**, (Left) Meta-analysis of human subcutaneous adipose tissue (SAT) and BAT proteome data (Müller et al., PMID:27418403). Heatmap showing relative protein levels of BAT-enriched genes. (Right) Heatmap showing relative protein levels of human BAT-enriched proteins in the proteome of WT and *Rnf20*$^{+/-}$ mice. **c,** Heatmap showing relative expression levels of eWAT-enriched genes. (Right) Heatmap showing relative protein levels of eWAT-enriched genes in the proteome of WT and *Rnf20*$^{+/-}$ mice. **d**, (Left) Heatmap showing relative protein levels of SAT-enriched genes. (Right) Heatmap showing relative protein levels of human SAT-enriched proteins in the proteome of WT and *Rnf20*$^{+/-}$ mice. **e**, Heatmap showing relative levels of proteins related to lipid storage and lipid droplet in BAT proteome of WT and *Rnf20*$^{+/-}$ mice. **f,** Heatmap showing relative levels of proteins that are suggested to be positively related to LD size (Min et al., PMID:31420514) in BAT proteome of WT and *Rnf20*$^{+/-}$ mice. **g**, Crucial transcription factors (TFs) of the downregulated proteins in BAT of *Rnf20*$^{+/-}$ mice. Data were analyzed by gene regulatory network based on the TF binding information. **h**, Gene network analysis with downregulated proteins in BAT of WT and *Rnf20*$^{+/-}$ mice. Target genes of the top five upstream regulators are indicated, and PPARγ target genes were labeled as blue. Color intensity is gradually darkened as the fold changes of protein levels between WT and *Rnf20*$^{+/-}$ mice. **i**, qRT-PCR analysis of PPARγ target genes in BAT of WT and *Rnf20*$^{+/-}$ mice housed at RT. $n$ = 5–7. **j**, **k**, qRT-PCR and Western blotting analysis of *Ncor1* in BAT of WT and *Rnf20*$^{+/-}$ mice housed at RT. $n$ = 5–7. **l**, **m**, qRT-PCR and Western blotting analysis of *Ncor1* in BAT of BAT Mock and BAT RNF20 OE mice housed at RT. $n$ = 4–6. **n**, **o**, qRT-PCR and Western blotting analysis of *Ncor1* in BAT siNC and BAT si*Rnf20* mice housed at RT. $n$ = 4–6. Source data are provided as a Source Data file. $n$ denotes biological replicates. Ct indicates critical value of qRT-PCR analysis. Data are represented as mean ± S.D. Significance (q value) was determined using multiple unpaired t-test (**i, j, l, n**). Full gene list was provided in Statistical source file.

# Supplementary Fig. 6 | GABPα Potentiates Thermogenesis in BAT.

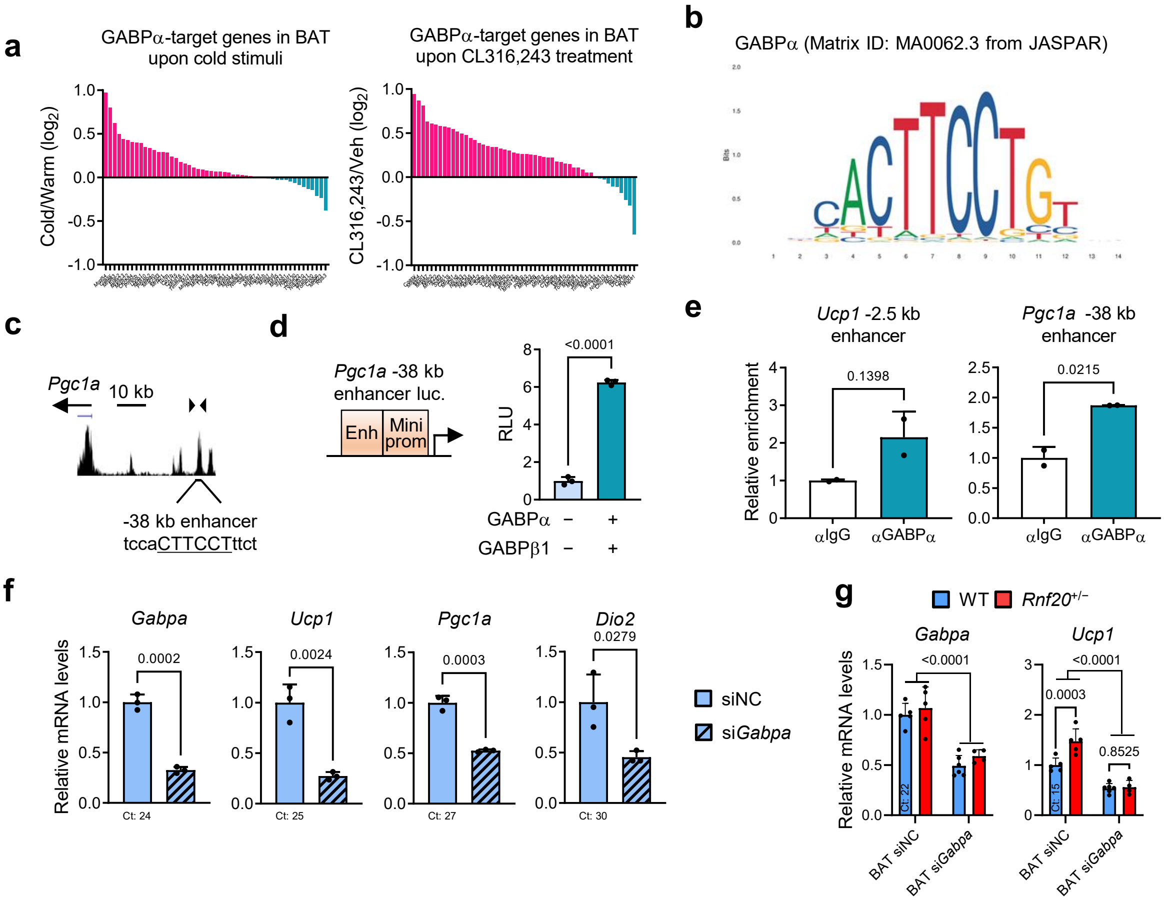


**Supplementary Fig. 6 | GABPα Potentiates Thermogenesis in BAT. Related to Fig. 3. a,** Expression levels of GABPα target genes in BAT upon cold stimuli or CL administration. Data from GSE51080 and GSE98132, respectively. **b,** GABPα binding motif matrix from JASPAR. **c**, Mouse *Pgc1a* enhancer regions with H3K27Ac enrichment peaks in BAT (GSE63964). The primers used for enhancer cloning are indicated. **d**, Luciferase activity of *Pgc1a*-luciferase (luc.) constructs in which their enhancer and minimal promoter (Mini prom) and TATA-box element are contained. $n = 3$. **e**, ChIP qRT-PCR assays of the *Ucp1* and *Pgc1a* enhancers in BAT. Enhancer occupancies by GABPα and IgG control were determined by ChIP qRT-PCR assays. $n = 2$. **f**, qRT-PCR analysis of differentiated brown adipocytes transfected with siNC or si*Gabpa*. $n = 3$. **g**, qRT-PCR assays of BAT of WT, *Rnf20*$^{+/-}$, WT with si*Gabpa*, and *Rnf20*$^{+/-}$ with si*Gabpa* mice housed under RT. $n = 4$–6. Source data are provided as a Source Data file. *n* denotes biological replicates. Ct indicates critical value of qRT-PCR analysis. Data are represented as mean ± S.D. Significance was determined using unpaired Student t-test (**d–f**) and two-way ANOVA (**g**).

# Supplementary Fig. 7 | In iWAT, RNF20 Potentiates Beige Adipocyte Thermogenesis upon Chronic Cold Stimuli

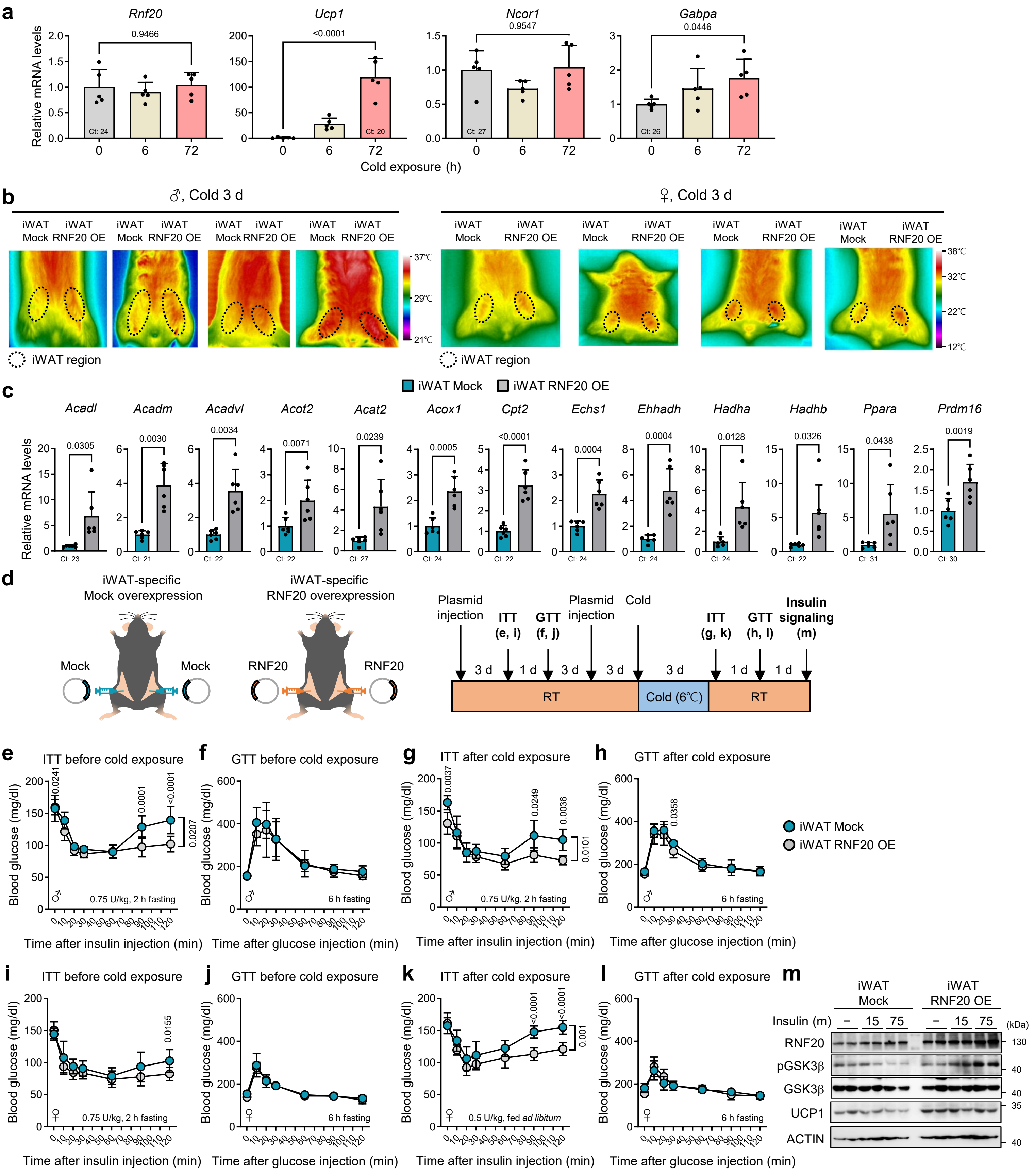


**Supplementary Fig. 7 | In iWAT, RNF20 Potentiates Beige Adipocyte Thermogenesis upon Chronic Cold Stimuli. Related to Fig. 5. a,** mRNA levels of *Rnf20*, *Ucp1*, *Ncor1*, and *Gabpa* in iWAT of mice exposed to cold (6°C) for 6 h and 3 d. **b,** Infrared camera images of male and female mice of iWAT-specific Mock and RNF20 OE mice exposed to cold for 3 d. **c**, mRNA levels of fatty acid oxidation and thermogenic genes in iWAT of iWAT-specific Mock and RNF20 OE mice exposed to cold for 3 d. $n$ = 6. **d**, Experimental scheme for iWAT-specific RNF20 overexpression (5 µg). ITT: insulin tolerance test, GTT: glucose tolerance test. **e–l**, Blood glucose levels during ITT and GTT. **m**, Western blotting analysis of iWAT from iWAT-specific Mock and RNF20 OE mice after cold exposure for 3 d. Source data are provided as a Source Data file. $n$ indicates biological replicates. Ct indicates critical value of qRT-PCR analysis. Data are represented as mean ± S.D. Significance was determined using one-way ANOVA (**a**), unpaired Student's t-test (**c**) and repeated-measures ANOVA (**e–l**).

# Supplementary Fig. 8 | In iWAT, RNF20 Potentiates Beige Adipocyte Thermogenesis upon Chronic Cold Stimuli, Independent of GABPα

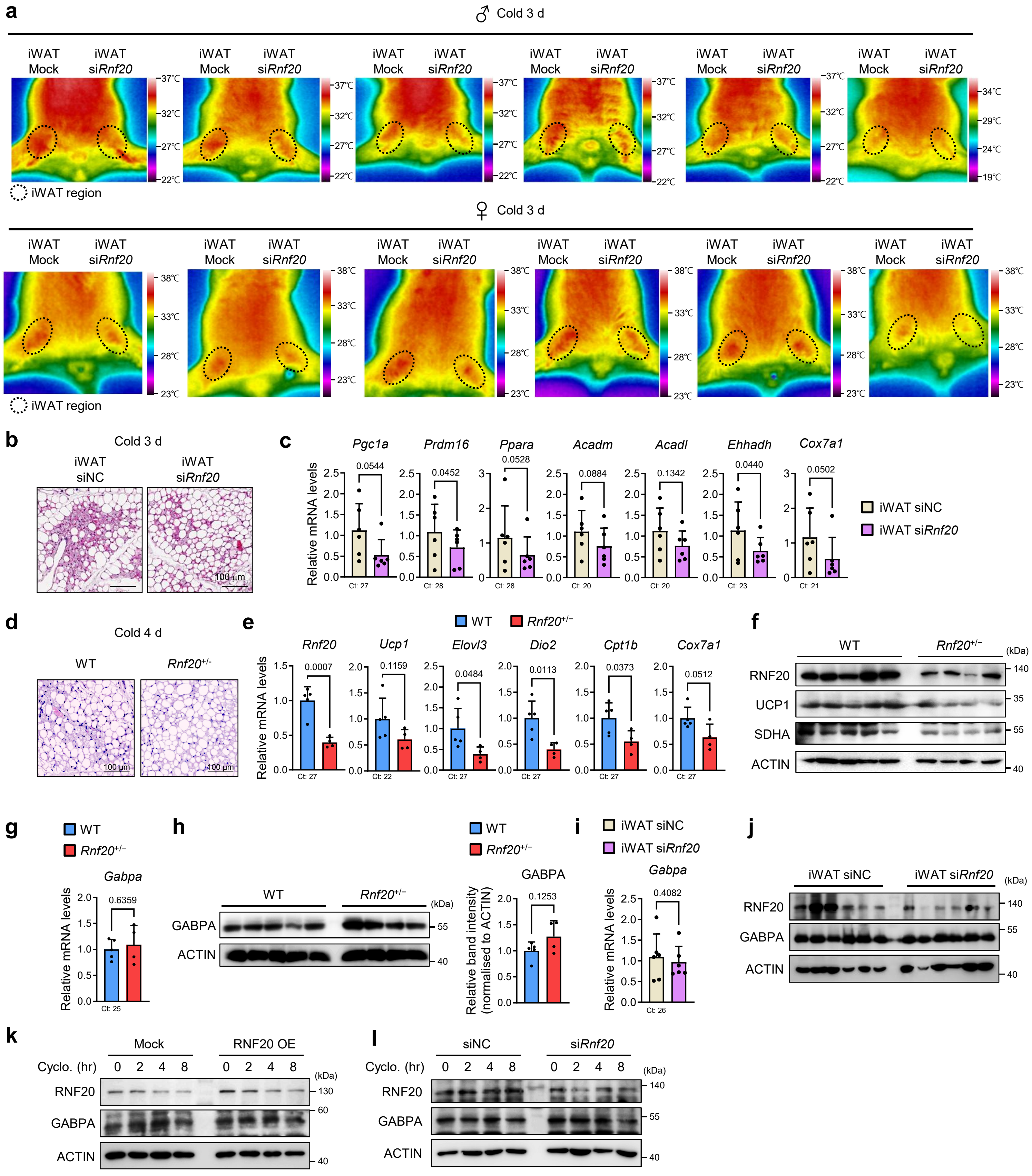


**Supplementary Fig. 8 | In iWAT, RNF20 Potentiates Beige Adipocyte Thermogenesis upon Chronic Cold Stimuli, Independent of GABPα. Related to Fig. 5. a**, Infrared camera images of male mice of iWAT-specific RNF20 KD. **b**, **c**, Representative H&E staining and mRNA levels of fatty acid oxidation and thermogenic genes in iWAT of iWAT-specific siNC and si*Rnf20* mice exposed to cold for 3 d. *n* = 6. **d–f**, Representative H&E staining, qRT-PCR and western blotting analyses of iWAT from WT and $Rnf20^{+/-}$ male mice exposed to cold for 4 d. *n* = 4–5. **g**, **h**. GABPα mRNA and protein level in iWAT from WT and $Rnf20^{+/-}$ male mice exposed to cold for 4 d. **i**, **j**. GABPα mRNA and protein level in iWAT from iWAT-specific siNC and si*Rnf20* mice exposed to cold for 3 d. **k**, **l**, Cycloheximide (30 μM)-chasing assay of GABPα protein in differentiated beige adipocytes from iWAT PDGFRα-expressing preadipocytes. Source data are provided as a Source Data file. *n* indicates biological replicates. Ct indicates critical value of qRT-PCR analysis. Data are represented as mean ± S.D. Significance was determined using paired Student's t-test (**c**, **i**) and unpaired Student's t-test (**e**, **g**, **h**).

# Supplementary Fig. 9 | iWAT RNF20 Potentiates Beige Fat Thermogenesis by Suppressing NCoR1 to Stimulate PPARγ

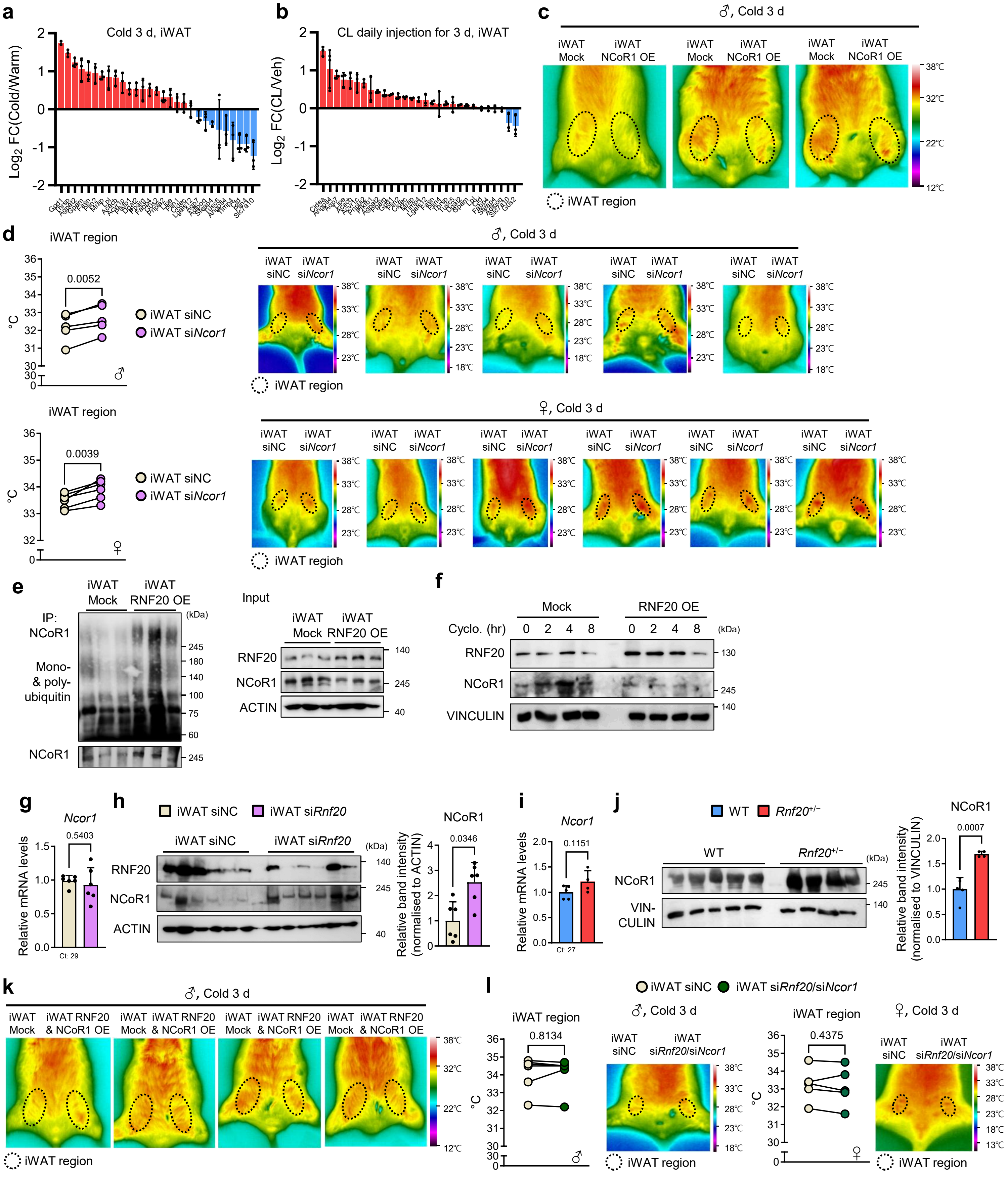

**Supplementary Fig. 9 | iWAT RNF20 Potentiates Beige Fat Thermogenesis by Suppressing NCoR1 to Stimulate PPARγ. Related Fig. 6. a, b,** Expression levels of PPARγ target genes in iWAT upon cold stimuli or CL administration. Data from GSE51080 and GSE98132, respectively. **c,** Infrared camera images of male mice of iWAT-specific NCoR1 OE. **d,** iWAT region temperatures and infrared camera images of male and female mice of iWAT-specific siNC and si*Ncor1* mice. **e,** Ubiquitin levels of NCoR1 in iWAT of iWAT-specific Mock and RNF20 OE male mice. **f,** Cycloheximide (30 μM)-chasing assay of GABPα protein in differentiated beige adipocytes from iWAT PDGFRα-expressing preadipocytes (for f, rosiglitazone was absent 2 days in the maintenance media). **g, h,** mRNA and protein levels of *Ncor1* in iWAT from iWAT-specific siNC and si*Rnf20* male mice exposed to cold for 3 d. **i, j,** mRNA and protein levels of *Ncor1* in iWAT from WT and $Rnf20^{+/-}$ male mice exposed to cold for 4 d. **k,** Infrared camera images and iWAT region temperatures of male mice of iWAT-specific RNF20&NCoR1 OE (**k**) and male and female mice of iWAT-specific si*Rnf20*/si*Ncor1* (**l**). Source data are provided as a Source Data file. *n* indicates biological replicates. Ct indicates critical value of qRT-PCR analysis. Data are represented as mean ± S.D. Significance was determined using paired Student t-test (**d, g, h, l**) and unpaired Student t-test (**i, j**).

# Supplementary Fig. 10 | iWAT RNF20 Potentiates *De novo* Beige Adipogenesis by Stimulating PPARγ

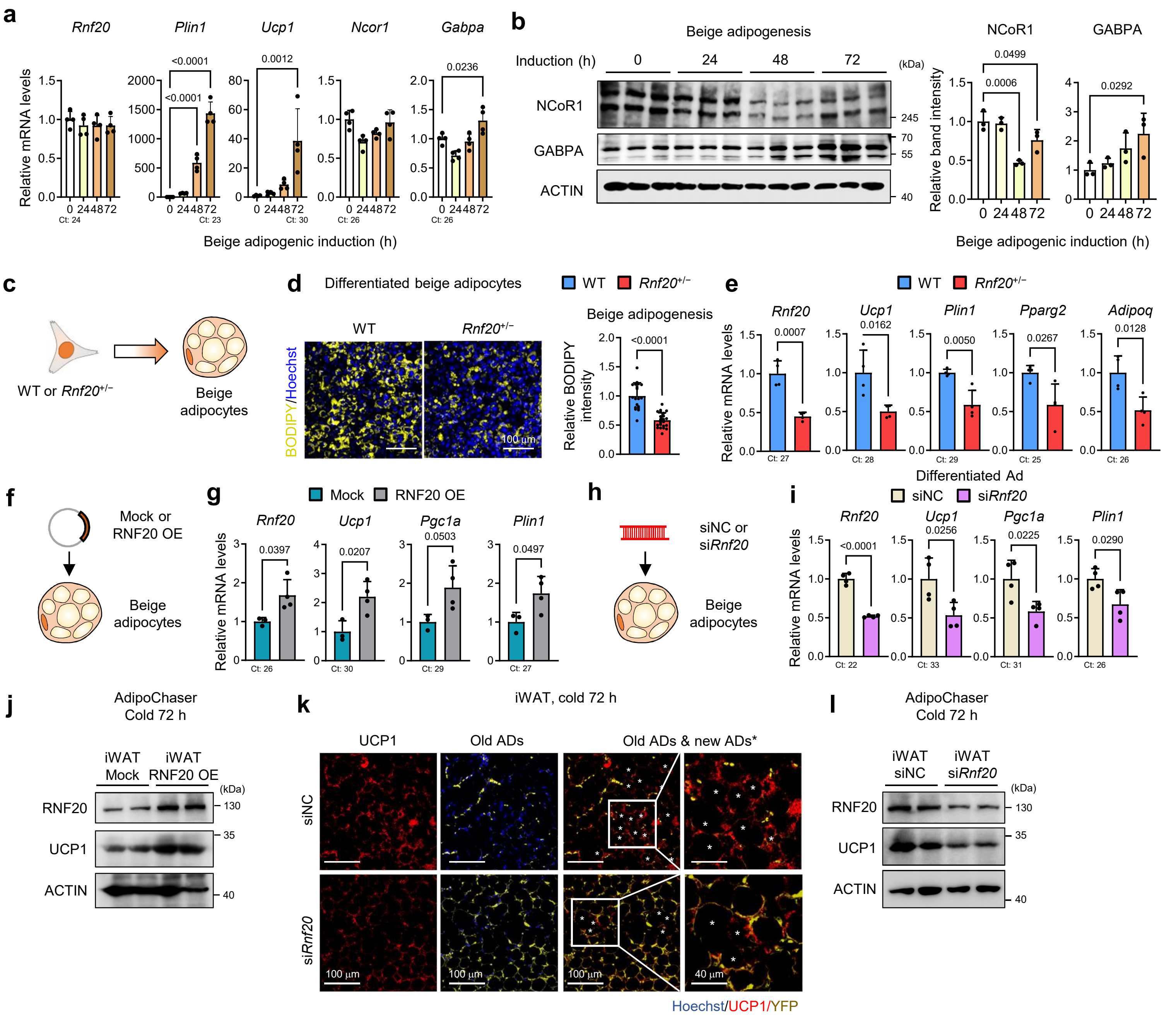

**Supplementary Fig. 10 | iWAT RNF20 Potentiates *De novo* Beige Adipogenesis by Stimulating PPARγ. Related Fig. 7. a, b,** qRT-PCR and Western blotting analysis of beige adipocytes differentiated from iWAT CD31$^-$CD45$^-$PDGFRα-expressing preadipocytes during adipogenesis. **c–e,** Experimental scheme, a representative image of BODIPY lipid staining and qRT-PCR analysis of differentiated beige adipocytes from WT and *Rnf20*$^{+/-}$ iWAT PDGFRα-expressing preadipocytes. **f–h**, Experimental scheme and qRT-PCR analysis of differentiated beige adipocytes without rosiglitazone in maintenance media and transfected with RNF20-expressing plasmids (**f, g**) or si*Rnf20* (**h**, **i**). **j**, Western blotting analysis of iWAT from iWAT-specific Mock and RNF20 OE AdipoChaser mice upon cold exposure (6°C) for 3 d. **k**, **l**, Representative whole-mount images of iWAT and Western blotting analysis of iWAT from iWAT-specific siNC and si*Rnf20* AdipoChaser mice upon cold exposure (6°C) for 3 d. The asterisk (*) refers to newly differentiated YFP-negative adipocytes. AD. Source data are provided as a Source Data file. *n* indicates biological replicates. Ct indicates critical value of qRT-PCR analysis. Data are represented as mean ± S.D. Significance was determined using ANOVA (**a**, **b**), unpaired Student t-test (**d, e, g, i**).

# Supplementary Fig. 11 | GABPα Polyubiquitination Is Increased by GABPα Overexpression in iWAT

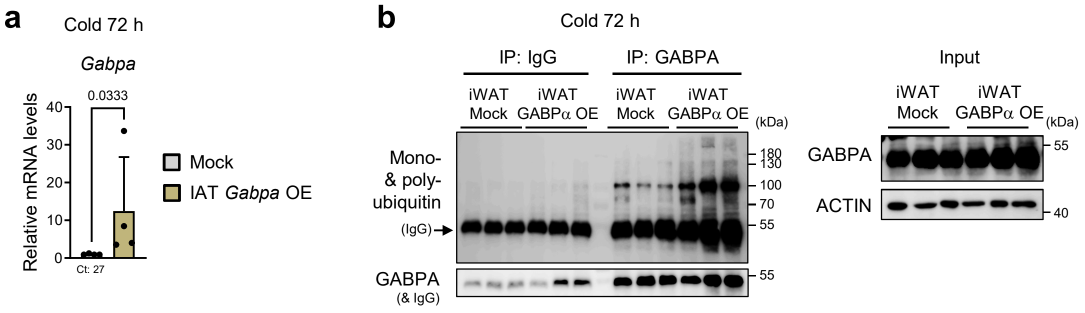


**Supplementary Fig. 11 | GABPα Polyubiquitination Is Increased by GABPα Overexpression in iWAT. Related Fig. 4 and 5. a,** mRNA levels of *Gabpa* in iWAT from iWAT-specific Mock and GABPα OE male mice exposed to chronic cold. **b**, Ubiquitin levels of GABPα in iWAT of iWAT-specific Mock and GABPA OE male mice exposed to chronic cold. Source data are provided as a Source Data file. *n* indicates biological replicates. Ct indicates critical value of qRT-PCR analysis. Data are represented as mean ± S.D. Significance was determined using paired Student t-test (**a**).
