# Supplementary material for: Ubiquitin ligase RNF20 coordinates sequential adipose thermogenesis with brown and beige fat-specific substrates
Source: Nat Commun. 2024 Jan 31;15:940. doi: 10.1038/s41467-024-45270-7 (PMC10831072; doi:10.1038/s41467-024-45270-7)
Supplement: Supplementary file 3 — Description of Additional Supplementary Files [file 41467_2024_45270_MOESM3_ESM.pdf]

Description of Additional Supplementary Files

Supplementary Data 1.

Sheet 1: Oligonucleotide sequences.

Sheet 2: BAT proteomics data of wild-type and *Rnf20* heterozygous-null mice housed under room temperature.
