# Supplementary material for: Ubiquitin ligase RNF20 coordinates sequential adipose thermogenesis with brown and beige fat-specific substrates
Source: Nat Commun. 2024 Jan 31;15:940. doi: 10.1038/s41467-024-45270-7 (PMC10831072; doi:10.1038/s41467-024-45270-7)
Supplement: Supplementary file 5 — Reporting Summary [file 41467_2024_45270_MOESM5_ESM.pdf]

nature portfolio

Corresponding author(s): Jae Bum Kim

Last updated by author(s): Jan 15, 2024

Reporting Summary

Nature Portfolio wishes to improve the reproducibility of the work that we publish. This form provides structure for consistency and transparency in reporting. For further information on Nature Portfolio policies, see our Editorial Policies and the Editorial Policy Checklist.

Statistics

For all statistical analyses, confirm that the following items are present in the figure legend, table legend, main text, or Methods section.

n/a	Confirmed	
[ ]	[x]	The exact sample size (*n*) for each experimental group/condition, given as a discrete number and unit of measurement
[ ]	[x]	A statement on whether measurements were taken from distinct samples or whether the same sample was measured repeatedly
[ ]	[x]	The statistical test(s) used AND whether they are one- or two-sided<br>*Only common tests should be described solely by name; describe more complex techniques in the Methods section.*
[x]	[ ]	A description of all covariates tested
[ ]	[x]	A description of any assumptions or corrections, such as tests of normality and adjustment for multiple comparisons
[ ]	[x]	A full description of the statistical parameters including central tendency (e.g. means) or other basic estimates (e.g. regression coefficient) AND variation (e.g. standard deviation) or associated estimates of uncertainty (e.g. confidence intervals)
[ ]	[x]	For null hypothesis testing, the test statistic (e.g. *F*, *t*, *r*) with confidence intervals, effect sizes, degrees of freedom and *P* value noted<br>*Give P values as exact values whenever suitable.*
[x]	[ ]	For Bayesian analysis, information on the choice of priors and Markov chain Monte Carlo settings
[x]	[ ]	For hierarchical and complex designs, identification of the appropriate level for tests and full reporting of outcomes
[ ]	[x]	Estimates of effect sizes (e.g. Cohen's *d*, Pearson's *r*), indicating how they were calculated

Our web collection on statistics for biologists contains articles on many of the points above.

Software and code

Policy information about availability of computer code

Data collection	The gene regulatory network was generated based on the transcription factor (TF) binding information from the ENCODE TF ChIP-seq (2015) and ChEA (2016) datasets retrieved from the Enrichr gene-set library and STRING database v11.5.
Data analysis	GraphPad Prism v10.0.2, STRING database v11.5, SciPy library v1.4.1, Cytoscape v3.8.2, Seurat v3.0.2 R package, Image J, MaxQuant software, Zen, LAS X, Bio-Rad CFX Maestro, FlowJo

For manuscripts utilizing custom algorithms or software that are central to the research but not yet described in published literature, software must be made available to editors and reviewers. We strongly encourage code deposition in a community repository (e.g. GitHub). See the Nature Portfolio guidelines for submitting code & software for further information.

Data

Policy information about availability of data

All manuscripts must include a data availability statement. This statement should provide the following information, where applicable:

- Accession codes, unique identifiers, or web links for publicly available datasets
- A description of any restrictions on data availability
- For clinical datasets or third party data, please ensure that the statement adheres to our policy

Proteomics data are provided in Supplementary Table 2. The used public data were GSE119452 for BAT RNA-seq, GSE125269 for BAT scRNA-seq, GSE63964 for BAT ChIP-seq, GSE68544 for brown adipocyte microarray, GSE179385 for iWAT RNA-seq, GSE98132 for WAT and BAT RNA-seq, and human adipose tissue proteome (PMID: 31420514). We also used the gene regulatory network was generated based on the transcription factor (TF) binding information from the ENCODE TF ChIP-

seq (2015) and ChEA (2016) datasets retrieved from the Enrichr gene-set library and STRING database v11.5.

Research involving human participants, their data, or biological material

Policy information about studies with human participants or human data. See also policy information about sex, gender (identity/presentation), and sexual orientation and race, ethnicity and racism.

Reporting on sex and gender	No human participants in this study
Reporting on race, ethnicity, or other socially relevant groupings	No human participants in this study
Population characteristics	No human participants in this study
Recruitment	No human participants in this study
Ethics oversight	No human participants in this study

Note that full information on the approval of the study protocol must also be provided in the manuscript.

Field-specific reporting

Please select the one below that is the best fit for your research. If you are not sure, read the appropriate sections before making your selection.

[x] Life sciences [ ] Behavioural & social sciences [ ] Ecological, evolutionary & environmental sciences

For a reference copy of the document with all sections, see nature.com/documents/nr-reporting-summary-flat.pdf

Life sciences study design

All studies must disclose on these points even when the disclosure is negative.

Sample size	The required sample size was determined based on previous preliminary experiments. For instance, in the rectal temperature measurement experiment, with a 95% confidence level and an allowable error of 2°C, using the standard deviation of 2°C obtained from the preliminary experiments, we calculated that n = (1.96)^2 * (2^2/2^2) = 3.84 mice are needed. Considering that an additional 10% of the sample size is required for preliminary purposes, we aimed to use a minimum of 4-5 mice per group. In the same line, the in vitro cell culture experiments were designed to have a minimum of n = 4.
Data exclusions	Mice with severe weight loss or hypothermia were excluded in consultation with the person in charge of animal testing.
Replication	All experiments were carried out under standard and clearly defined conditions. All attempts at replication were successful by at least one researcher
Randomization	Age and gender matched mice were randomly allocated to experimental groups.<br>For cell culture experiments, cells were randomly assigned to experimental groups.
Blinding	Animal experiments were performed blindly for control and treatment groups.

Reporting for specific materials, systems and methods

We require information from authors about some types of materials, experimental systems and methods used in many studies. Here, indicate whether each material, system or method listed is relevant to your study. If you are not sure if a list item applies to your research, read the appropriate section before selecting a response.

Materials & experimental systems

n/a	Involved in the study	
[ ]	[x]	Antibodies
[ ]	[x]	Eukaryotic cell lines
[x]	[ ]	Palaeontology and archaeology
[ ]	[x]	Animals and other organisms
[x]	[ ]	Clinical data
[x]	[ ]	Dual use research of concern
[x]	[ ]	Plants

Methods

n/a	Involved in the study	
[x]	[ ]	ChIP-seq
[ ]	[x]	Flow cytometry
[x]	[ ]	MRI-based neuroimaging

Antibodies

Antibodies used	Antibodies against RNF20 (ab32629; Abcam), UCP1 (ab10983; Abcam) TUBULIN (T6199; Sigma-Aldrich), TOMM20 (612272; BD Biosciences), SDHA (CSB-PA01985A0Rb; Cusabio), PPARg (sc-7196; Santa Cruz Biotechnology), PLIN1 (20R-PP004; Fitzgerald), MYC-tag (05-724, Millipore), FLAG-tag (F1804; Sigma-Aldrich), NCoR1 (ab3482; Abcam), adiponectin (2789; Cell Signaling Technology), b-ACTIN (A5316; Sigma-Aldrich), GABPA (MA5-15419; Invitrogen), Mono- and polyubiquitinylated conjugates (ENZ-ABS840; Enzo Life Sciences), GSK3b (610201; BD bioscience), phospho-GSK3b (9336; Cell Signaling Technology), OXPHOS (ab110413; Abcam), rabbit IgG (A0545; Sigma-Aldrich), mouse IgG (A9044; Sigma-Aldrich), CD31-FITC (102405, BioLegend), CD45-FITC (103107, BioLegend), and PDGFRa-PE (135905, BioLegend)
Validation	RNF20 antibody (Abcam Cat# ab32629; RRID: AB_873630)<br>https://www.abcam.com/products/primary-antibodies/rnf20-antibody-ab32629.html<br>Host Organism: rabbit<br>Clonality: polyclonal<br>Dilution: 1:1000<br><br>UCP1 antibody (Abcam Cat# ab10983; RRID: AB_2241462)<br>https://www.abcam.com/products/primary-antibodies/ucp1-antibody-ab10983.html<br>Host Organism: rabbit<br>Clonality: polyclonal<br>Dilution: 1:1000<br><br>TUBULIN antibody (Sigma-Aldrich Cat# T6199; RRID: AB_477583)<br>https://www.sigmaaldrich.com/KR/ko/product/sigma/t6199<br>Host Organism: mouse<br>Clonality: monoclonal<br>Dilution: 1:1000<br><br>TOMM20 antibody (BD Biosciences Cat# 612278; RRID: AB_399595)<br>https://www.bdbiosciences.com/en-eu/products/reagents/microscopy-imaging-reagents/immunofluorescence-reagents/purified-mouse-anti-tom20.612278<br>Host Organism: mouse<br>Clonality: monoclonal<br>Dilution: 1:1000<br><br>SDHA antibody (Cusabio Cat# CSB-PA01985A0Rb)<br>https://www.cusabio.com/Polyclonal-Antibody/SDHA-Antibody-172659.html<br>Host Organism: rabbit<br>Clonality: polyclonal<br>Dilution: 1:1000<br><br>PPARg antibody (Santa Cruz Biotechnology Cat# sc-7196, RRID:AB_654710)<br>https://www.scbt.com/p/ppargamma-antibody-h-100<br>Host Organism: rabbit<br>Clonality: polyclonal<br>Dilution: 1:1000<br><br>PLIN1 antibody (Fitzgerald Industries International Cat# 20R-PP004, RRID:AB_1288416)<br>https://fitzgerald-fii.com/perilipin-antibody-20r-pp004.html<br>Host Organism: guinea pig<br>Clonality: polyclonal<br>Dilution: 1:1000<br><br>MYC antibody (Millipore Cat# 05-724, RRID:AB_309938)<br>https://www.merckmillipore.com/KR/ko/product/Anti-Myc-Tag-Antibody-clone-4A6,MM_NF-05-724?ReferrerURL=https%3A%2F%2Fwww.google.com%2F<br>Host Organism: mouse<br>Clonality: monoclonal<br>Dilution: 1:1000<br><br>FLAG antibody (Sigma-Aldrich Cat# F1804, RRID:AB_262044)<br>https://www.sigmaaldrich.com/KR/ko/product/sigma/f1804<br>Host Organism: mouse<br>Clonality: monoclonal<br>Dilution: 1:1000<br><br>NCoR1 antibody (Abcam Cat# ab3482, RRID:AB_10860296)<br>https://www.abcam.com/products/primary-antibodies/nuclear-receptor-corepressor-ncor-antibody-ab3482.html

Host Organism: rabbit
Clonality: polyclonal
Dilution: 1:1000

Adiponectin antibody (Cell Signaling Technology Cat# 2789, RRID:AB_2221630)
https://www.cellsignal.com/products/primary-antibodies/adiponectin-c45b10-rabbit-mab/2789?_requestid=1201187
Host Organism: rabbit
Clonality: monoclonal
Dilution: 1:1000

ACTIN antibody (Sigma-Aldrich Cat# A5316, RRID:AB_476743)
https://www.sigmaaldrich.com/KR/ko/product/sigma/a5316l
Host Organism: mouse
Clonality: monoclonal
Dilution: 1:1000

GABPA antibody (Thermo Fisher Scientific Cat# MA5-15419, RRID:AB_10977231)
https://www.thermofisher.com/antibody/product/GABPA-Antibody-clone-8C1B10-Monoclonal/MA5-15419
Host Organism: mouse
Clonality: monoclonal
Dilution: 1:1000 for WB, 1:200 for IP

Mono- and polyubiquitinylated conjugates antibody (Enzo Life Sciences Cat# ENZ-ABS840, RRID:AB_2935893)
https://www.enzolifesciences.com/ENZ-ABS840/mono-and-polyubiquitinylated-conjugates-recombinant-monoclonal-antibody-ubcj2/
Host Organism: mouse
Clonality: recombinant monoclonal
Dilution: 1:1000

GSK3b antibody (BD Biosciences Cat# 610201, RRID:AB_397600)
https://www.bdbiosciences.com/en-us/products/reagents/microscopy-imaging-reagents/immunofluorescence-reagents/purified-mouse-anti-gsk-3.610201
Host Organism: mouse
Clonality: monoclonal
Dilution: 1:1000

Phospho-GSK3b antibody (Cell Signaling Technology Cat# 9336, RRID:AB_331405)
https://www.cellsignal.com/products/primary-antibodies/phospho-gsk-3b-ser9-antibody/9336
Host Organism: rabbit
Clonality: polyclonal
Dilution: 1:1000

OXPHOS antibody (Abcam Cat# ab110413, RRID:AB_2629281)
https://www.abcam.com/products/panels/total-oxphos-rodent-wb-antibody-cocktail-ab110413.html
Host Organism: mouse
Clonality: monoclonal
Dilution: 1:1000

Vinculin antibody (Cell Signaling Cat#4650, RRID:AB_10559207)
https://www.cellsignal.com/products/primary-antibodies/vinculin-antibody/4650
Host Organism: rabbit
Clonality: polyclonal
Dilution: 1:1000

Anti-mouse IgG antibody (Santa Cruz Biotechnology Cat#sc-2025; RRID:
https://www.scbt.com/p/normal-mouse-igg
Host Organism: mouse
Clonality: polyclonal
Dilution: 1:200 for IP

Anti-Rabbit IgG peroxidase antibody (Sigma-Aldrich Cat# A0545; RRID:AB_257896)
https://www.sigmaaldrich.com/US/en/product/sigma/a0545
Host Organism: goat
Clonality: polyclonal
Dilution: 1:5000 for secondary antibody

Anti-Mouse IgG peroxidase antibody (Sigma-Aldrich Cat# A9044; RRID: AB_258431)
https://www.sigmaaldrich.com/US/en/product/sigma/a9044
Host Organism: rabbit

Clonality: polyclonal
Dilution: 1:5000 for secondary antibody

FITC anti-mouse CD31 antibody (BioLegend Cat# 102405, RRID:AB_312900)
https://www.biolegend.com/en-gb/neuroscience-1/fitc-anti-mouse-cd31-antibody-120
Host Organism: rat
Clonality: monoclonal
Dilution: 1:200 for FACS

FITC anti-mouse CD45 antibody (BioLegend Cat# 103107, RRID:AB_312972)
https://www.biolegend.com/en-us/products/fitc-anti-mouse-cd45-antibody-99?GroupID=BLG1932
Host Organism: rat
Clonality: monoclonal
Dilution: 1:200 for FACS

PE anti-mouse CD140a (PDGFRa) antibody (BioLegend Cat# 135905, RRID:AB_1953268)
https://www.biolegend.com/en-gb/products/pe-anti-mouse-cd140a-antibody-6253
Host Organism: rat
Clonality: monoclonal
Dilution: 1:100 for FACS

Eukaryotic cell lines

Policy information about cell lines and Sex and Gender in Research

Cell line source(s): Immortalized murine brown preadipocytes (BACs), HEK293T cells

Authentication: Immortalized BAC cells were provided by Dr. Kai Ge (National Institutes of Health). HEK293T cells were authenticated by ATCC. Additionally, we verified the characteristics of the cell lines using species- and cell-type-specific primers and morphology.

Mycoplasma contamination: All cell lines were tested negative for mycoplasma contamination

Commonly misidentified lines (See ICLAC register): No commonly misidentified lines were applied in this study

Animals and other research organisms

Policy information about studies involving animals; ARRIVE guidelines recommended for reporting animal research, and Sex and Gender in Research

Laboratory animals: 3-4-month-old C57BL/6J male and female mice were used. In Rnf20 defective (Rnf20+/−) mice, exons 3–20 of the Rnf20 gene were deleted. Rnf20+/− mice were obtained from the knockout mouse project repository (KOMP). This mouse strain, [C57BL/6N-Rnf20tm1 (KOMP)Vlcg/TcpMmucd (RRID:MMRRC_049486-UCD)], was obtained from the Mutant Mouse Resource and Research Center at the University of California at Davis. Mice were housed in a temperature- and humidity-controlled, specific pathogen-free animal facility at 22°C, under a 12:12 h light:dark cycle, and health status checks were performed two or three times a week.

Wild animals: No wild animals were used in the study.

Reporting on sex: We performed animal experiments with both male and female mice.

Field-collected samples: No field-collected samples were used in the study.

Ethics oversight: The animal study and experimental procedures were approved by the Seoul National University Institutional Animal Care and Use Committee.

Note that full information on the approval of the study protocol must also be provided in the manuscript.

Flow Cytometry

Plots

Confirm that:

- [x] The axis labels state the marker and fluorochrome used (e.g. CD4-FITC).
- [x] The axis scales are clearly visible. Include numbers along axes only for bottom left plot of group (a 'group' is an analysis of identical markers).
- [x] All plots are contour plots with outliers or pseudocolor plots.
- [x] A numerical value for number of cells or percentage (with statistics) is provided.

Methodology

Sample preparation	Inguinal adipose tissues were minced and digested with collagenase buffer [0.1 M HEPES,0.125 M NaCl, 5 mM KCl, 1.3 mM CaCl2, 5 mM glucose, 1.5% (w/v) bovine serum albumin, and 0.1% (w/v) collagenase I (Worthington, 49A18993)] in a shaking water bath at 37C for 30–60 min. After centrifugation at 200 3 g, room temperature (RT) for 5 min, the pelleted stromal vascular fraction (SVF) was collected. The SVF was incubated in red blood cell lysis buffer (1.7 M Tris, pH 7.65, and 0.16 M NH4Cl) for 15 min. Then, the SVFs were washed with phosphate-buffered saline (PBS) several times, passed through a 100-um filter (93100, SPL), and collected by centrifugation at 200 x g for 5 min. SVFs were incubated with FACS primary antibodies for 30 min at 4°C and were gently washed with and resuspended in PBS. Then, the cells were filtered with Falcon® Round-Bottom Tubes with Cell Strainer Cap, 5 mL.
Instrument	FACS Canto II instrument (BD Biosciences)
Software	FlowJo
Cell population abundance	Frequencies of cell populations were determined based on contour plots provided in Supplementary Information
Gating strategy	Singlets were gated on FSC-A vs FSC-H and live cells were defined using FSC-A vs SSC-A. iWAT adipose stem cells were gated as CD31-CD45-PDGFRa+.

☒ Tick this box to confirm that a figure exemplifying the gating strategy is provided in the Supplementary Information.
